# A Novel Amdoparvovirus of Badgers and Foxes and the Perpetuation of Aleutian Mink Disease Virus 3 in the Wildlife of Denmark

**DOI:** 10.3390/pathogens14080734

**Published:** 2025-07-25

**Authors:** Frederikke Juncher Høeg, Anne Sofie Vedsted Hammer, Anna Cecilie Boldt Eiersted, Joost Theo Petra Verhoeven, Lars Erik Larsen, Tim Kåre Jensen, Marta Canuti

**Affiliations:** 1 Department of Veterinary and Animal Sciences, University of Copenhagen, 1870 Frederiksberg, Denmarktkje@sund.ku.dk (T.K.J.); 2 Centre for Evolutionary Hologenomics, The Globe Institute, University of Copenhagen, 1353 Copenhagen, Denmark; joost.verhoeven@sund.ku.dk

**Keywords:** amdoparvovirus carnivoran, wild animals, virus ecology, mink farming, carnivore, virus discovery

## Abstract

Amdoparvoviruses, encompassing the well-characterized Aleutian mink disease viruses (AMDV) as well as less investigated viruses infecting both captive and wild animals, are important carnivoran viruses that are significant pathogens in the mink farming industry. We investigated the molecular epidemiology of amdoparvoviruses among Danish wildlife. Spleen samples from 118 animals of seven carnivoran species were screened with a pan-amdoparvovirus PCR, and the identified viruses were molecularly characterized. In one of five European badgers (*Meles meles*), we identified an AMDV-3 strain whose ancestors were likely of farmed mink origin. This virus was last reported on a mink farm in 2002, demonstrating how farm-derived viruses have established themselves among wildlife. We also discovered and fully characterized a novel virus found in five of 81 (6.2%) foxes (*Vulpes vulpes*) and one of five badgers (20.0%), which we named fox and badger amdoparvovirus 1 (FBAV-1). FBAV-1 fulfills the criteria for classification as a novel species and phylogenetically is positioned as an intermediate between the North American and Eurasian amdoparvoviral clades. This study provides baseline data and expands our understanding of amdoparvoviral ecology. Further studies including more animals across diverse geographic areas are warranted to clarify amdoparvovirus epidemiology, spread, cross-species transmission, epidemic potential, and evolutionary paths.

## 1. Introduction

Amdoparvoviruses (viruses within the genus *Amdoparvovirus* of the family *Parvoviridae*) are non-enveloped viruses characterized by a single-stranded DNA (ssDNA) monosense genome. The genome is linear, with a size of ~4 kb and includes two main gene cassettes—one for non-structural (NS) proteins (NS1-3) and one for capsid proteins (VP1-2)—flanked by imperfect palindromic sequences that fold into hairpin-like structures [1,2]. The NS1 contains the highly conserved superfamily 3 (SF3) helicase domain, which is essential for viral replication. Unlike other parvoviruses, VP1 of amdoparvoviruses lacks the phospholipase A2 (PLA2) domain, typically involved in endosomal escape [3,4]. The VP proteins are highly immunogenic, and anti-VP antibodies have been implicated in antibody-dependent enhancement (ADE) of infection [4,5,6,7]. Due to their key functional role in the infection cycle, amdoparvoviral VP proteins, unlike those of other parvoviruses, are highly conserved, even more than the NS proteins [4]. Nonetheless, like for all other parvoviruses, taxonomic classification is based on the NS1 protein, with an identity cut-off for species demarcation of 85% [1,2]. *Amdoparvovirus* currently includes 11 classified species, 10 of which comprise viruses of carnivorans (animals within the order Carnivora) [1,4].

One of the most well-studied viruses in the genus is Aleutian mink disease virus (AMDV), known to cause large epidemics with high mortalities in domesticated mink [3,8,9,10]. The various AMDV lineages (AMDV1-3 and British Columbia amdoparvovirus, BCAV) belong to multiple viral species, specifically *Amdoparvovirus carnivoran1* and *Amdoparvovirus carnivoran8-10* [1,4]. Among wild animals, AMDV has been identified predominantly in mustelids (American and European mink, martens, and ermines) and their predators (e.g., fox and lynx) [3,4,11,12,13]. In adult mink, AMDV gives rise to persistent infections and causes Aleutian disease (AD, also referred to as plasmacytosis), an immune complex-related condition characterized by hypergammaglobulinemia, lymphadenopathy, splenomegaly, weight loss, lethargy, anemia, and renal involvement. In severe or late-stage diseases, immune complex deposition in the capillaries of organs can lead to glomerulonephritis, hepatic necrosis, vasculitis, and eventually death [3,14,15,16,17,18]. AMDV infections are also associated with reduced pregnancy rates, decreased litter size, embryonic death, and abortion [3,19]. Mink farms infected with imported (non-autochthonous) variants of AMDV can act as a source of infection for local wildlife, posing a threat to local animal populations [3,13,18,20,21,22,23]. Mink farming has been the source of introduction of both American mink and AMDV into wildlife populations in many different countries. As a result, although AMDV originated in North America, the various virus species and variants have a global distribution [4,8,21,24].

Skunk amdoparvovirus (SKAV, *Amdoparvovirus carnivoran4*) is widely distributed across North America, where it infects skunks and, occasionally, sympatric mink [12,25,26,27]. Unlike AMDVs, in the absence of anthropogenic animal movement, viruses within this species strongly segregate geographically [28]. Furthermore, other carnivoran amdoparvoviruses have been detected in a range of wild and captive carnivorans. These include five viruses found in foxes, comprising the grey fox amdoavirus (GFAV) [29], the red fox fecal amdovirus (RFFAV) [30], the raccoon dog and fox amdoparvovirus (RFAV) [31], and the Labrador amdoparvovirus (LaAV) 1 and 2 [11]. Additionally, there have been reports of divergent amdoparvoviruses in ferrets [32,33], and recent studies detected amdoparvoviruses in red pandas [34,35,36], lynx [37] and badgers, including the meles meles amdoparvovirus (MMAV) [38], the European mustelid amdoparvovirus 1 (EMAV-1) [37], and an unnamed virus [13]. These findings highlight the broad host range of these viruses and suggest unrecognized viral diversity within the genus.

Infections caused by some amdoparvoviruses have been associated with clinical and pathological signs similar to AMDV, suggesting that similar mechanisms of immune-mediated pathology occur across the genus [28,29,36,39,40,41,42,43,44]. Similarly, most amdoparvoviruses of carnivorans are known to lack strict host specificity, and this is likely linked to their ability to infect circulating macrophages, one of their target cells, through antibodies, without the need for a specific receptor [4]. Cross-species transmission has indeed been frequently observed for several of these viruses, including AMDV and SKAV [20,25], but also LaAV-1, which can infect foxes and martens [11], RFAV, which can cause infection in raccoon dogs, foxes, mink, and badgers [4,38] and EMAV-1, which was found in badgers and stone martens [37]. Such host plasticity raises important questions about the transmission dynamics of amdoparvoviruses in the wild. While spillover and maintenance within multi-host systems are likely, it is crucial to understand the mechanisms enabling these events, especially in shared ecological contexts and across trophic levels.

In Europe, AMDV has been extensively reported in farmed mink [8,10,24,45,46,47,48,49,50,51] and occasionally also in wild mink and other mustelids, suggesting spillover and possible maintenance in wildlife populations [23,52,53]. Additionally, a novel amdoparvovirus has been recently identified in Estonian Eurasian badgers [13]. Also, RFFAV, EMAV-1, and EFAV-1 were described in the Iberian Peninsula [30,37], supporting the view that amdoparvoviruses may be endemic in multiple European carnivoran populations. However, amdoparvoviral diversity, host range, and geographic distribution are still not well understood, and for several geographic regions, it is currently unknown which are the autochthonous amdoparvoviruses. Finally, no studies have explored these viruses in detail in Denmark. Therefore, the objective of this study was to identify which amdoparvoviruses are circulating in populations of wild terrestrial carnivorans in Denmark in order to assess viral host and geographic dispersal as well as molecularly characterize the identified viruses to increase our understanding of the ecology of amdoparvoviruses in the unexplored European carnivoran reservoir.

## 2. Materials and Methods

### 2.1. Study Locations, Samples, and Data Collection

**Figure 1:**
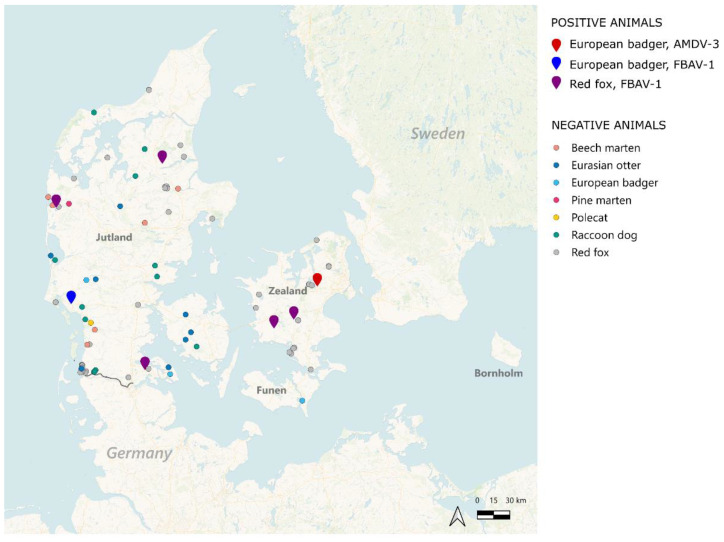
Sample collection locations across Denmark. The map shows the locations of sample collection, which are indicated by colored circles, while pins indicate amdoparvovirus-positive animals, as indicated in the legend. AMDV-3: Aleutian mink disease virus 3 (AMDV-3); FBAV-1: fox and badger amdoparvovirus 1.

This project included 118 archived spleen samples previously (2018–2024) harvested from wild carnivorans, hunted or road-killed throughout Denmark (Figure 1), within the context of the annual Danish national passive wildlife disease surveillance program, performing disease surveillance throughout the country. Denmark is located in Northern Europe and is the southernmost of the Scandinavian countries. It is formed by the northern part of the Jutland peninsula and an archipelago of over 400 islands, of which the largest is Zealand, where the capital, Copenhagen, is situated. Among others, the island of Funen is located between Jutland and Zealand, while the island of Bornholm is located ~150 km east of the rest of the country, in the Baltic Sea (Figure 1). The animals included in this study came from Jutland (N = 77), Funen (N = 4), and Zealand (N = 24).

Tissue sampling was performed by experienced veterinary pathologists during routine necropsy procedures, and the samples were collected only from animals in adequate post-mortem condition (i.e., not showing signs of advanced decomposition). During sampling, sterile, single-use instruments and containers were employed to avoid cross-contamination, and all tissue samples were stored at −80 °C in a dedicated tissue archive until further analysis. Carnivorans included in this study comprised 81 red foxes (*Vulpes vulpes*), 13 raccoon dogs (*Nyctereutes procyonoides*), 10 Eurasian otters (*Lutra lutra*), 6 beech martens (*Martes foina*), 5 European badgers (*Meles meles*), 2 pine martens (*Martes martes*), and 1 polecat (*Mustela putorius*). The majority of fox cadavers, obtained through hunting or roadkill, were collected as part of a national surveillance program for *Echinococcus multilocularis*. To ensure broad geographic coverage, efforts were made to involve collaborators from all regions of the country, including personnel from the regional units of the Danish Nature Agency. In contrast, mustelid carcasses—species for which no hunting season exists—were primarily submitted through the national passive wildlife disease surveillance program and were therefore not geographically targeted. All available samples from the tissue archive were included in the study without any selection or exclusion criteria. Sex was recorded for 88.1% (104/118) of the animals, and 52.9% (55/104) of these were males, and 47.1% (49/104) were females. The approximate age was known for 76.3% (90/118) of individuals, and 95.6% (86/90) of them were adults. Necropsy reports, including weight at the time of recovery, were available for all animals.

### 2.2. Molecular Methods

DNA was isolated from ~10 mg of each tissue using the DNeasy Blood & Tissue Kit (Qiagen, Hilden, Germany), according to the manufacturer’s instructions. The extracted DNA was eluted in 100 μL of elution buffer and stored at −80 °C until analysis. A previously published pan-amdoparvovirus heminested PCR [11,37], designed to amplify a highly conserved region of the VP gene and capable of detecting known as well as novel viruses within the genus *Amdoparvovirus*, was used to perform pan-amdoparvovirus molecular screening. A positive control, an AMDV-G (*Amdoparvovirus carnivoran1*) positive cell culture supernatant, was included in all tests. The additional primer AMDK_1F (GTMACAGAAA-CTAACCAAGGC) was used to perform a second heminested PCR on the product amplified from the first pan-amdoparvovirus PCR to obtain full fragment amplification and sequencing. All samples were also screened for the presence of canine parvovirus type 2 (CPV-2) and feline panleukopenia virus (FPV) [54] to assess possible co-infections. Screening primer sequences are available in Table S1. Additional primers (sequences available upon request) were designed based on sequences obtained in this study and others available in GenBank and used to amplify and sequence larger genomic fragments. Amplicons were purified with the HighPrep PCR-DX purification beads (MAGBIO Genomics, Gaithersburg, MD, USA) and outsourced for Sanger sequencing.

### 2.3. Sequence and Phylogenetic Analyses

Following Sanger sequencing, primer sequences and low-quality ends were trimmed from the obtained reads, which were then assembled to form a single sequence with Geneious Prime (Dotmatics, Bishop’s Stortford, UK). Geneious was also used to predict open reading frames (ORFs) and splicing sites, based on available data on other amdoparvoviruses [3], which were also confirmed with NNSPLICE 0.9 [55]. Amino acid sequences were then inferred from the nucleotide sequences. The reference sequences for phylogenetic analyses were downloaded from GenBank and imported into Geneious to create multiple sequence alignments using MAFFT [56]. Following multiple sequence alignments, the nucleotide alignments were exported and used to build maximum-likelihood phylogenetic trees using IQTree2 [57]. The ModelFinder function [58] was used to perform a ModelTest analysis for each alignment to identify the best-fit model for genetic distance estimation. Shimodaira–Hasegawa approximate likelihood-ratio test (SH-alrt) and ultrafast bootstrapping (1000 replicates) were performed for statistical verification [59,60]. The phylogenetic trees were annotated with INKSCAPE 1.4 (www.inkscape.org, accessed on 1 January 2025).

### 2.4. Statistical Analysis and Maps

Percentages with 95 % normal intervals (95% intervals of confidence, 95% IC) were used to express categorical variables, while continuous variables were expressed in medians with interquartile ranges (IQR, 25th–75th percentile). Fisher’s exact test was used to assess associations between categorical variables, and the Mann–Whitney U test was applied to median comparisons; two-sided *p*-values < 0.05 were considered statistically significant. Statistical analyses were performed with PAST version 4.17 (https://www.nhm.uio.no/english/research/resources/past/, accessed on 1 January 2025). 

The map visualization was performed in QGIS version 3.42.3 (www.qgis.org, accessed on 1 January 2025) using map data provided by OpenStreetMap (openstreetmap.org/copyright). The map style used was provided by CartoDB Inc. under the CC-BY 4.0 license.

## 3. Results

### 3.1. Virus Epidemiology and Molecular Typing

Overall, 7 of the 118 animals (5.9%, 95% CI: 4.2–11.8%) showed evidence for amdoparvoviral infection, and viruses were found in red foxes and European badgers. None of the animals were CPV-2/FPV-positive. The prevalence of amdoparvoviruses among foxes was 6.2% (5/81, 95% CI: 2.0–13.8%), while two of the five badgers were positive (40.0%). Viral positivity did not significantly differ between foxes and badgers (*p* = 0.09). Among the positive animals, 71.4% (5/7) were females and 28.65% (2/7) were males, 85.7% (6/7) were adults, and for 1 positive animal, the age was unknown. Additionally, 42.8% (3/7) of them were sampled in Zealand, 28.6% (2/7) in the northern part of Jutland, and 26.6% (2/7) of the samples came from the southern part of Jutland (Figure 1). No significant difference in prevalence was observed between different demographic groups or geographic regions. Viral prevalence was the highest during the summer months, but the number of samples was highly variable across seasons, making statistical comparisons difficult (winter: 5/47% (10.6%, 95% CI: 3.5–23.1%), spring: 0/6, summer: 1/3 (33.3%, 95% CI: 0.8–90.6%), fall: 1/13 (7.7%, 95% CI: 0.2–22.2%)). The pathological reports revealed no gross signs of disease in positive animals. Among female foxes (the only group with a meaningful sample size, N = 44 with 4 positive animals), the weight did not differ significantly between infected and non-infected individuals (5827 (4541–6408) vs. 5683 (5206.5–6428), *p* = 0.5).

**Figure 2:**
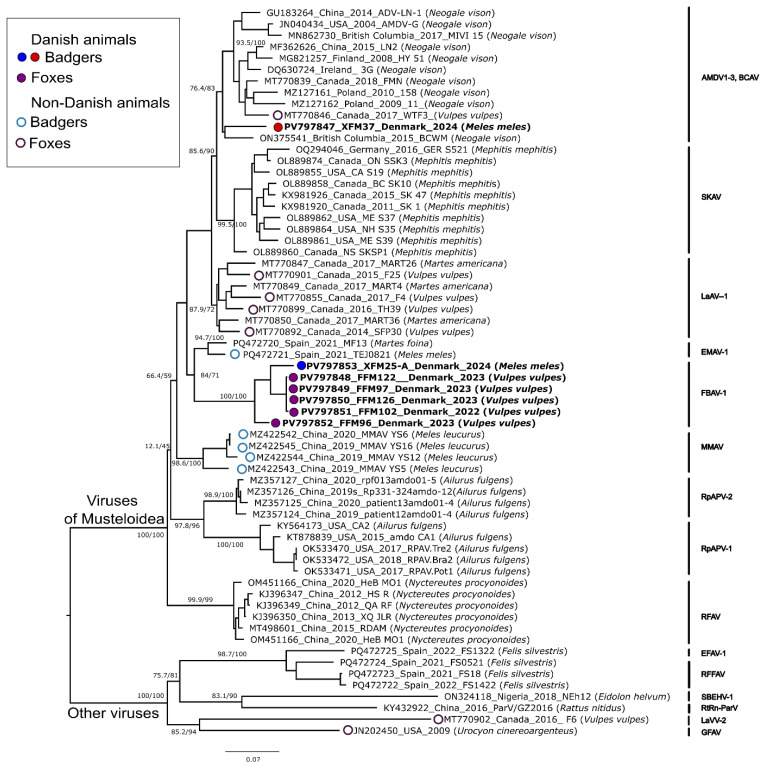
Phylogenetic analysis of amdoparvoviruses investigated in this study. The maximum likelihood tree was built from partial (~816 nt) VP sequences obtained from five foxes and two badgers found in this study, as well as 57 reference sequences. The phylogenetic tree was built based on the TVM + F + I + R3 model with IQ-tree. The outcomes of the SH-alrt and bootstrap tests (1000 replicates) are shown for the main nodes. The sequences of viruses found in foxes and badgers are labeled with colored circles corresponding to the animal in which they were found, according to the legend on the left. Sequences obtained in this study are highlighted in bold. When available, each sequence name includes the GenBank accession number, the sampling collection site and year, the viral variant name, and the animal species in which the virus was identified. Virus abbreviations are available in Table S2.

A ~800 nt-long fragment was sequenced for each of the seven positive samples to type the detected amdoparvoviruses, and the phylogenetic relationships between the identified strains and those from other hosts and locations were investigated. The obtained tree comprised two main clades. The first was a larger one containing viruses so far identified as having members of the Musteloidea as their maintenance hosts, which also included all sequences from this study. The second was a smaller one containing viruses from other hosts (canids, felids, bats, and rodents). In total, the phylogenetic tree could be divided into 15 clades, each corresponding to previously defined or proposed amdoparvoviral species or species groups (Figure 2). As viruses belonging to the various AMDV clades and BCAV cannot be distinguished from each other using this genomic region [4,37], they are shown as one clade in the tree. One of the seven sequenced viruses (XFM37), which was from Zealand, originated from a badger and was located in the AMDV/BCAV clade. Conversely, the remaining six viruses found in this study formed a highly supported (bootstrap = 100, SH-alrt = 100) independent clade. Within this clade, five viruses were found in foxes and one in a badger, and the animals originated from Northern and Southern Jutland as well as from Zealand. The pairwise sequence identity between the viruses within this clade ranged between 94.4 and 100% at the nucleotide level and between 91.6 and 100% at the protein level. The closest relatives to these viruses were variants of EMAV-1, a virus recently discovered in Spanish martens and badgers [37], with which they formed a highly supported clade (bootstrap = 71, SH-alrt = 84). The six divergent study sequences were <91% identical to any other amdoparvovirus in this genomic region. Similar values could be observed for pairwise sequence identities between viruses from different defined species. Therefore, it was concluded that these sequences likely represented a new viral species. The virus was named fox and badger amdoparvovirus 1 (FBAV-1).

### 3.2. Taxonomical Investigation of Fox and Badger Amdoparvoviruses

**Figure 3:**
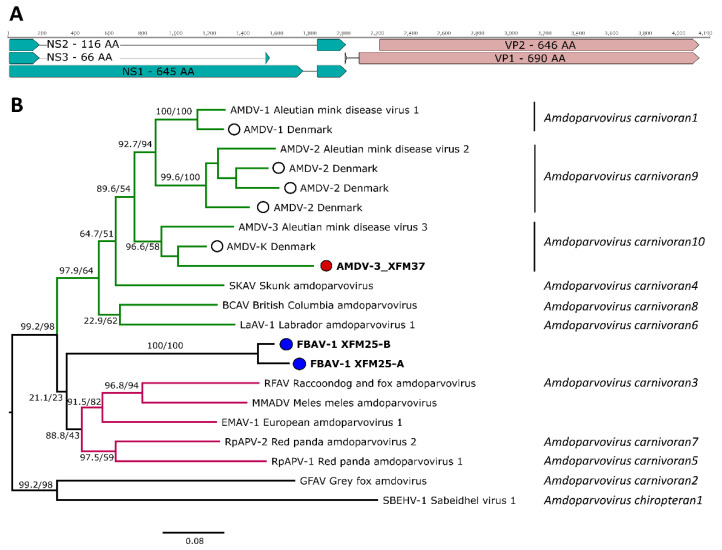
Genome organization, phylogenetic analysis, and taxonomy of detected amdoparvoviruses. The genome organization of strain XFM25-A is shown in panel (**A**). Splicing patterns and ORFs for the non-structural proteins and structural proteins are shown in green and pink, respectively. Protein sizes as well as names are indicated. The maximum likelihood tree (panel (**B**)) was constructed from full NS1 protein sequences obtained in this study compared to reference sequences from each classified and completely characterized amdoparvoviral species and representatives of Danish strains (Table S2). The clade including viruses from North America is indicated in green, and the one including viruses from Eurasia in pink. Viruses from this study are indicated with colored circles, while other Danish sequences are indicated with empty circles. Phylogenetic inference was conducted using the Q.yeast + I + R3 substitution model as implemented in IQ-TREE2. Node support is indicated at nodes and was calculated by SH-aLRT and ultrafast bootstrapping (1000 replicates).

To characterize the identified viruses, we attempted to obtain their full genomic sequences. Unfortunately, the low viral load, the high divergence from reference sequences, and multiple infections in the same animals limited our outcomes. While terminal repeat sequences were not determined, we were able to obtain the full coding sequence of the AMDV strain from badger XFM37 and of one FBAV-1 variant from the positive badger (XFM25-A) and a complete NS gene cassette of another variant identified in the same badger (XFM25-B). The predicted splicing pattern of XFM37 was comparable to those of other AMDVs, while the one of the two FBAV-1 variants was more similar to the splicing profile of EMAV-1 and MMAV, resulting in a two-amino-acid insertion at the acceptor site of NS1 (Figure 3A). Typical amdoparvoviral NS1 protein motifs were observed in all viruses: rolling circle replication motifs II (HIH in both viruses) and III (YLFNKDK for AMDV and YLFNKEK for FBAV-1), and the helicase domains Walker domains A (GPGGTGKTL), B (IWAEE), B′ (KAITGGGDVKVDTKNKQPQ), and C (VLVTSN for AMDV and VIVTSN for FBAV-1).

To determine the taxonomy of the detected amdoparvoviruses, we performed a phylogenetic analysis based on full NS1 protein sequences. The three NS1 ORFs were obtained after *in silico* splicing, and the derived amino acid sequences were aligned with those of previously characterized amdoparvoviruses retrieved from GenBank (Table S2). In the resulting phylogenetic tree (Figure 3B), XFM-37 clustered within a well-supported clade corresponding to AMDV-3, including the Danish strain AMDV-K, and belonged to the species *Amdoparvovirus carnivoran10*. In contrast, the sequences XFM-25A and XFM-25B formed a distinct clade, genetically separated from all so far recognized amdoparvoviruses, including the various AMDV variants previously identified in Denmark. Interestingly, in the clade of viruses from Musteloidea, we could distinguish a clade consisting of sequences from North America (green branches) and one of sequences from Eurasia (pink branches). The sequences found in this study did not belong to either of those clades and formed a third middle clade.

According to the ICTV classification criteria, parvoviruses are considered to belong to the same species if their NS1 proteins share more than 85% amino acid sequence identity. Pairwise identity analysis between XFM-25A/B and other amdoparvoviruses revealed identity values below 76% for NS1 and 93% for VP2. The NS1 proteins of XFM25A and XFM25B were 94.3% identical to each other. Thus, the identified FBAV-1 variants represent the first discovered members of a potential new species within the genus *Amdoparvovirus*.

As in this analysis we could not compare the obtained sequences to those identified in Estonian badgers [13] and those obtained from Japanese and American ferrets [32,33], as they were only partially sequenced, two separate analyses were performed (Figures S1 and S2). FBAV-1 sequences were only 62.4–66.7% identical to the sequences from Estonia (NS1) and 87.9–89% identical to viruses identified in ferrets (VP). Additionally, Estonian and ferret sequences clustered independently and separately from FBAV-1 in a clade including RFAV and EMAV-1, respectively. Therefore, we concluded that the Estonian and badger viruses do not belong to the same species as FBAV-1.

### 3.3. AMDVs in Denmark

**Figure 4:**
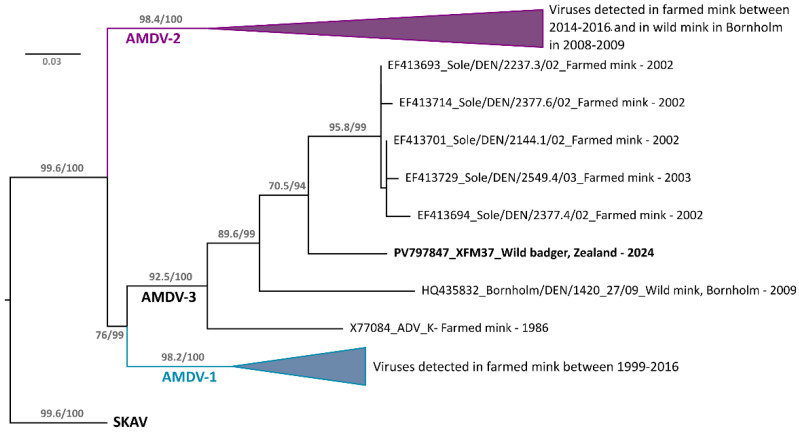
Phylogenetic analysis of Danish Aleutian mink disease viruses 1-3. The maximum likelihood tree was built from partial (~328 nt) NS1 nucleotide sequences obtained from Danish AMDV-1-3 sequences (N = 389) and two skunk amdoparvovirus (SKAV) sequences, used as an outgroup. The phylogenetic tree was built based on the TIM3 + F + I + R3 model with IQ-tree2. The outcome of the ultrafast bootstrap (1000 replicates) and the SH-aLRT tests are shown for the main nodes. The virus identified in this study (XFM37) is highlighted in bold. The clades containing AMDV-1 and AMDV-2 sequences were collapsed, and the clades are labeled at their nodes. Each sequence name includes the GenBank accession number, the viral variant name, the sampled animal (including whether it was from a wild animal or a mink farm), and the sampling year.

To investigate the origin of the AMDV-3 variant we identified in the wild badger from Zealand (XFM37), we downloaded from GenBank all Danish amdoparvoviral sequences (N = 423), including multiple sequences obtained throughout the years (1984–2016) from farmed mink [10,24,49,61,62] and several sequences obtained from wild mink sampled on the island of Bornholm [23]. A partial NS1 gene fragment that was represented by the vast majority of available sequences (N = 388) was then used to perform a comprehensive phylogenetic analysis (Figure 4). XFM37 was included in a small clade whose root was one of the very first sequenced AMDV strains, AMDV-K, which was isolated from Danish farmed mink kits with interstitial pneumonia in the 1980s [62,63]. The same clade included a few sequences connected to an epidemic in farmed mink from southern Jutland/Funen from 2002 [62] and one sequence from a wild mink from the island of Bornholm, sampled in 2009 [23]. Within the clade, XFM37 was the closest to the strains from 2002 (92.7–93.9%) and more loosely related to AMDV-K (88.7%) and the virus from the wild mink (89.0%). This was the smallest of the three clades and did not include any of the strains causing farm epidemics in more recent years (all included in AMDV-1 and AMDV-2 clades).

## 4. Discussion

Although AMDV has been known for over 50 years [3], other amdoparvoviruses have only been recently identified (2011–2025) [4,37,38]. Indeed, the scientific knowledge about these new viruses is limited, and their impact and spread, particularly among wild animals, are unknown. Therefore, we wanted to investigate these viruses in wildlife to improve our understanding of their distribution, diversity, ecology, and potential for cross-species transmission. For this purpose, 118 spleen samples collected from seven different carnivoran species sampled across Denmark were screened for amdoparvoviruses and the most widespread parvoviruses of carnivorans, CPV-2 and FPV. While none of the animals were positive for CPV-2/FPV, ~6% of the animals were amdoparvovirus-positive, and these viruses were identified in two out of seven species investigated, foxes and badgers. Although the presence of multiple infections and low DNA concentrations made sequencing challenging, some sequence information was obtained from all identified viruses, including two almost complete genomes.

### 4.1. A Novel Amdoparvovirus of Carnivorans

Most of the identified viruses formed a separate clade within genus-wide phylogenies, and the genomic and pairwise sequence identity analysis indicated that these viruses are the first discovered members of a potential new species. Indeed, the NS1 of virus variants within this clade fulfilled the 85% identity criterion for species demarcation [1,2]. Although its host range might be wider, we decided to name this virus fox and badger amdoparovirus 1 (FBAV-1) to reflect the two animal hosts in which it was first identified, consistent with the nomenclature of other amdoparvoviruses. The prevalence of FBAV-1 in foxes was approximately 6%, while 20% of the investigated badgers were FBAV-1-positive. However, the difference in positivity between the two species was not statistically significant and may be attributed to the disparity in sample sizes (81 vs. 5, with only one positive badger detected).

These results indicated that FBAV-1 is capable of cross-species transmission, as also previously reported for other amdoparvoviruses [4,11]. This is probably associated with the fact that amdoparvoviruses have the ability to infect macrophages of different host species without requiring a specific receptor for cell entry [4]. While FBAV-1 was identified in two different host species, it is not possible to conclude which one is the main maintenance host, but there are several possibilities. First, the virus could be circulating in both foxes and badgers. The second possibility is trophic transmission, meaning that foxes and badgers may have become infected while feeding on animals of an unknown species, which could be the main host of this virus, as it can occur for AMDV, which can be transmitted from mink to its predators [11,64]. The third possibility is that the virus primarily infects only badgers or foxes, and that other species occasionally become infected through direct or indirect contact, as it has also been reported for other amdoparvoviruses [11,12,65]. Studies have documented social interactions between different carnivoran species and, particularly, between foxes and badgers. These animals are, in fact, frequently observed together, and it has been reported that badgers share dens with foxes and raccoon dogs; it is even possible for animals from all three species to occupy the same den simultaneously [64,66]. This behavior could be a possible cause of direct transmission between these animals. Interestingly, however, none of the 13 investigated raccoon dogs tested positive for FBAV-1.

Other amdoparvoviruses were found in foxes in previous studies, but they all belonged to different viral species, including RFFAV in Spain [30], GFAV in the USA [29], and AMDV, LaAV-1, and LAV-2 in Canada [11]. Interestingly, most of these viruses belong to the amdoparvoviral clade that does not include viruses of Musteloidea, while LaAV-1 and FBAV-1, included within the Musteloidea infecting virus clade, were also identified in martens and badgers, respectively. Likewise, amdoparvoviruses were also found in badgers in previous studies, including MMAV in China [38], EMAV-1 in Spain [37], and an unnamed virus related to RFAV in Estonia [13]. All these viruses are included in the bigger clade of viruses of Musteloidea. While further epidemiological studies are required to confirm this hypothesis, we can speculate that FBAV-1 is originally a virus of badgers that can spill over to foxes, as we also previously hypothesized for LaAV-1 [11].

FBAV-1 was detected across Denmark (both in Northern and Southern Jutland as well as Zealand), indicating that the virus is disseminated throughout the country. It would be interesting to collect and test additional samples from other locations, including other countries in Northern Europe, to investigate further the host and geographic distribution of this virus and compare it to that of the virus found in Estonian badgers. Additionally, all samples investigated in this study were from recent years, and investigating older archived samples could help elucidate epidemiological trends of this virus throughout the years.

Finally, the badger and foxes that tested positive for FBAV-1 appeared healthy at the time of necropsy, and no significant decrease in weight, indicative of a wasting disease, was observed. However, these conclusions have to be regarded as preliminary, and it remains plausible that microscopic pathological alterations detectable only through histological examination were present. It also still needs to be established whether this virus can cause chronic infections, like other amdoparvoviruses do [3,4]. Indeed, out of the five positive foxes, one case of co-infection by two different variants was identified, and this is a typical feature of amdoparvoviruses causing persistent infections, facilitating superinfections of different variants [3,25,67]. Certainly, follow-up studies involving more individuals with a wider range of ages and more targeted post-mortem examinations will be required to clarify the pathogenic role of this virus.

### 4.2. The Perpetuation of AMDV-3 in the Wildlife of Denmark

The American mink (*Neogale vison*), native to North America, is an alien species in Europe and was brought to Denmark (including to the Island of Bornholm) in the 1920s–1930s for the establishment of fur farms [68]. Animal trading within the context of fur farming was also responsible for the introduction in Eurasia of various amdoparvoviruses [47], likely also of North American origin [3,4,21]. These viruses, which were collectively known as AMDV (although later on it was established that they belonged to multiple species [4]) leaked from farms and started circulating among feral American mink, their descendants, and local wildlife [13,21,53,69].

In this study, we identified an ADMV-3 (*Protoparvovirus carnivoran10*) variant in a badger from Zealand (XFM37). The oldest Danish report and sequence of AMDV-3 is the strain AMDV-K, which was isolated from an outbreak involving 3 mink farms in 1982 [63]. While most of the more recent (2004–2016) epidemics in Danish mink farms involved AMDV-1 (*Protoparvovirus carnivoran1*) and AMDV-2 (*Protoparvovirus carnivoran9*), a large (220 farms) AMDV-3 feed-born epidemic was reported in Southern Jutland and Funen in 2002 (strain Sole/DEN/02) [49]. This AMDV-3 variant is the closest known relative of XFM-37. Additionally, AMDV-3 was also reported once in wild mink from the remote island of Bornholm [23], in the only other published study about amdoparvoviruses in Danish wildlife. Interestingly, according to the annual wildlife casualty surveys reported to the Environmental Protection Agency in Denmark (www.patologivagten.dk, accessed on 5 May 2025), several animals have been regularly investigated by antibody testing or PCR for AMDVs, and badgers, beech martens, polecat, and mink were found to be positive. Unfortunately, these samples were not available for this research, and a future priority will be to expand sample collection and testing.

While it is impossible, at this point, to accurately identify the origin of XFM37, it is likely that ancestors of this virus were introduced in Denmark through animal import within the context of mink farming, spread to wildlife, and crossed the species barrier between mink and badger, and likely other mustelids. Given also the generally high mutation rate of parvoviruses [70,71], the virus we see today has likely diverged significantly from the original virus. Since Bornholm is separated from Zealand, where the AMDV-3-positive badger was sampled, by ~150 km of sea, it is unlikely that XFM37 is directly connected to viruses found in wild mink on this Island. However, it is possible that its ancestors are somehow connected to the farm outbreak of 2002. Taken together, these results confirm that AMDV strains are circulating among Danish wildlife, and multiple reservoir hosts that are responsible for spreading these viruses likely exist. A more extended and thorough investigation of Danish wildlife is required to elucidate the local history of these viruses and clarify epidemiological trends and paths of cross-species transmission. This is also important considering that mink farming is planned to be resumed in Denmark.

### 4.3. Final Considerations About the Ecology and Evolution of Amdoparvoviruses

While a correspondence between virus and host phylogenies, indicative of virus–host co-evolution, has been previously hypothesized [4], the recent amdoparvovirus discoveries are making this pattern less clear. While it is still true that viruses of canids and felids are more distant and cluster separately from viruses of mustelids, the bigger clade of viruses identified in members of the Musteloidea is becoming more and more “contaminated”. Specifically, some of these viruses were found in canids, even at high prevalence. In detail, RFAV has been found in sympatric farmed animals, including canids and mustelids [4,38], making it impossible to make a definitive assumption about its original wild host(s). LaAV-1 [11] and FBAV-1 were both found in canids (foxes) and mustelids (martens and badgers) and, while it is possible that mustelids were the original maintenance hosts for these viruses, available data are too scarce to make conclusive statements. It is nonetheless true that, as important biological viral features are conserved across the genus [4], cross-species transmission plays a crucial role in shaping the ecology and evolution of amdoparvoviruses.

Finally, in phylogenetic analyses, an interesting pattern could be observed in the “Musteloidea clade”. A sub-clade containing viruses originating from North America was observed that included the various farm-related AMDVs and BCAV, SKAV (USA and Canada), and LaAV-1 (Canada). Additionally, a clade of Eurasian viruses was present, including EMAV (Spain), MMAV (Chinese farms), RpAPV-1 and RpAPV-2 (viruses of red pandas found in several zoos), and RFAV (Asian farms). Interestingly, FBAV-1 did not belong to either of these clades. This is an indication of local drift and speciation in the absence of anthropogenic-related animal relocations and suggests that more amdoparvoviruses likely exist in unexplored areas.

Remarkably, viruses from ferrets, whose closest relatives were the viruses from Spanish mustelids, showed a pattern similar to AMDVs, as the same viral lineage was identified in America and Japan [32,33]. This possibly suggests that anthropogenic-related animal movements could be affecting the epidemiology of these viruses too. Nonetheless, the close relationship between the ferret and Spanish strains is extremely interesting and will have to be elucidated in future studies.

## 5. Conclusions

This study presents the molecular epidemiology of amdoparvoviruses detected in Danish wildlife. Although the number of analyzed samples and species was low, and the obtained sequencing information was limited, we were able to identify two viral species and draw meaningful conclusions about amdoparvoviral ecology. An AMDV-3 strain whose ancestors were likely of mink farm origin was found in a badger, highlighting how “old” amdoparvovirus strains that leaked from farms have established themselves among wildlife and can infect multiple hosts. This study also led to the discovery of the first members of a previously unknown viral species, FBAV-1, for which evidence for cross-species transmission was found as the virus was detected in foxes and badgers. While the extent of amdoparvovirus circulation remains undetermined, both these viruses are likely widespread in Northern Europe and across multiple hosts, potentially threatening the health of endangered populations. This study also provides baseline data about amdoparvovirus circulation in Danish wildlife, a particularly relevant aspect as mink farming is planned to be resumed in Denmark. It is essential in the future to investigate a larger number of animals from a wider area to thoroughly study amdoparvovirus spreading dynamics, cross-species transmission, epidemic potential, and evolutionary paths. Additionally, since some divergent viruses detected in wild animals have only been sporadically investigated and partially characterized (e.g., viruses from Spanish felids and canids, Estonian badgers, or domestic ferrets), future epidemiological investigations as well as complete genome sequencing are warranted to fully clarify virus movements across different host populations. It is also important to continue amdoparvovirus surveillance and full-genome sequencing, as many undiscovered viruses may still be circulating.

## Data Availability

Sequences obtained in this study were deposited in GenBank under accession numbers PV797847-PV797854.

## Supplementary Materials

The following supporting information can be downloaded at https://www.mdpi.com/article/10.3390/pathogens14080734/s1: Figure S1: Phylogenetic analysis comparing study viruses to those found in Estonian badgers; Figure S2: Phylogenetic analysis comparing study viruses to those found in ferrets; Table S1. Primers used for virus screening; Table S2: Reference sequences used to build the tree in Figure 4. Refs. [72,73] are cited in Supplementary Materials.

## Abbreviations

The following abbreviations are used in this manuscript:

ADE	Antibody-dependent enhancement
AMDV	Aleutian mink disease virus
CI	Confidence interval
CPV-2	Canine parvovirus type 2
DNA	Deoxyribonucleic acid
EFAV	European fox amdoparvovirus
EMAV	European mustelid amdoparvovirus
FBAV	Fox and badger amdoparvovirus
FPV	Feline panleukopenia virus
GFAV	Grey fox amdovirus
ICTV	International committee on taxonomy of viruses
IQR	Interquartile range
LaAV	Labrador amdoparvovirus
MMAV	Meles meles amdoparvovirus
NS1	Non-structural protein 1
ORF	Open reading frame
PCR	Polymerase chain reaction
QGIS	Quantum geographic information system
RFAV	Raccoon dog and fox amdoparvovirus
RFFAV	Red fox fecal amdovirus
SH-aLRT	Shimodaira–Hasegawa approximate likelihood-ratio test
SKAV	Skunk amdoparvovirus
ssDNA	Single-stranded DNA
VP	Virus (capsid) protein
