# Supplementary material for: A Novel Amdoparvovirus of Badgers and Foxes and the Perpetuation of Aleutian Mink Disease Virus 3 in the Wildlife of Denmark
Source: Pathogens. 2025 Jul 25;14(8):734. doi: 10.3390/pathogens14080734 (PMC12389654; doi:10.3390/pathogens14080734)
Supplement: Supplementary file 1 [file pathogens-14-00734-s001.zip › pathogens-3738982-supplementary.pdf]

A novel amdoparvovirus of badgers and foxes and the perpetuation of Aleutian mink disease virus 3 in the wildlife of Denmark

Frederikke Juncher Høeg, Anne Sofie Vedsted Hammer, Anna Cecilie Boldt Eiersted, Joost Theo Petra Verhoeven, Lars Erik Larsen, Tim Kåre Jensen, Marta Canuti

Supplementary Table S1. Primers used for virus screening

**Primer**	**Sequence (5′ – 3′)**
**Pan-amdoparvovirus screening** (Canuti et al., 2020a)	
P1F	CCAACAAGTAATGACACCWTGG
P2R	CTCCAGYAAAGTAACTACC and GTCCACCAACAAAGTAACTACC
P1R	GTTGGTTTRGTTGCTCTCC
Protoparvovirus carnivoran1 **screening** (Canuti et al., 2020b)	
CPV-2_ScF	GRGTGATGGAGCAGTWCAAC
CPV-2_ScR	CATCAACYAATGACCAAGGTG
CPV-2_ScF2	AACCAGACGGTGGTCARYC

Supplementary Table S2. Reference sequences used to build the tree in Figure 4

**Virus name**	**Abbreviation**	**Species name (if classified)**	**Accession number**	**Reference**
Aleutian mink disease virus 1	AMDV-1	*Amdoparvovirus carnivoran1*	M20036	(Canuti et al., 2022)
Aleutian mink disease virus 1 (from Denmark)	AMDV-1	*Amdoparvovirus carnivoran1*	KY996931	(Hagberg et al.,2017)
Aleutian mink disease virus 2 (from Denmark)	AMDV-2	*Amdoparvovirus carnivoran9*	KY997057<br>KY996952<br>KY996985	(Hagberg et al.,2017)
Aleutian mink disease virus 3 (from Denmark)	AMDV-3	*Amdoparvovirus carnivoran10*	X77084<br><br>PV797847	(Gottschalck et al., 1994)<br>(This study)
Grey fox amdovirus	GFAV	*Amdoparvovirus carnivoran2*	MG821261	(Li et al., 2011)
Raccoon dog and fox amdoparvovirus	RFAV	*Amdoparvovirus carnivoran3*	KY68028	(Shao et al., 2014)
Skunk amdoparvovirus	SKAV	*Amdoparvovirus carnivoran4*	KX981923	(Canuti et al., 2017)
Red panda amdoparvovirus 1	RpAPV-1	*Amdoparvovirus carnivoran5*	KT878839	(Alex et al., 2018)
Labrador amdoparvovirus 1	LaAV-1	*Amdoparvovirus carnivoran6*	MT770849	(Canuti et al., 2020a)
Red panda amdoparvovirus 2	RpAPV-2	*Amdoparvovirus carnivoran7*	MZ357124	(Zhao et al., 2022)
British Columbia amdoparvovirus	BCAV	*Amdoparvovirus carnivoran8*	ON375541	(Canuti et al., 2022)
Aleutian mink disease virus 2	AMDV-2	*Amdoparvovirus carnivoran9*	MG821261	(Canuti et al., 2022)
Aleutian mink disease virus 3	AMDV-3	*Amdoparvovirus carnivoran10*	KY680280	(Canuti et al., 2022)
Sabeidhel virus 1	SBEHV-1	*Amdoparvovirus chiropteran1*	ON324118	(Kamani et al., 2022)
Meles meles amdoparvovirus	MMADV	-	MZ422542	(Wu et al., 2024)
European amdoparvovirus 1	EMAV-1	-	PQ472720	(Canuti et al, 2025)
Fox and Badger Amdoparvovirus 1	FBAV-1	-	PV797853<br>PV797854	This study

Supplementary Figure S1. **Phylogenetic analysis comparing study viruses to those found in Estonian badgers.**

The maximum likelihood tree was constructed using partial NS1 amino acid sequences (93 aa) from representative amdoparvoviruses, including known AMDV-1, AMDV-2, and AMDV-3 variants, as well as other carnivoran-associated amdoparvoviruses. The tree was inferred using the Q.mammal+G4 substitution model in IQ-TREE2, with branch support assessed by 1,000 ultrafast bootstrap replicates and SH-aLRT tests. The newly identified viruses from Denmark (XFM-37, XFM-25A, and XFM-25B) are shown in bold and labelled with a colored circle. Viruses identified in Estonian badgers are highlighted in bold.

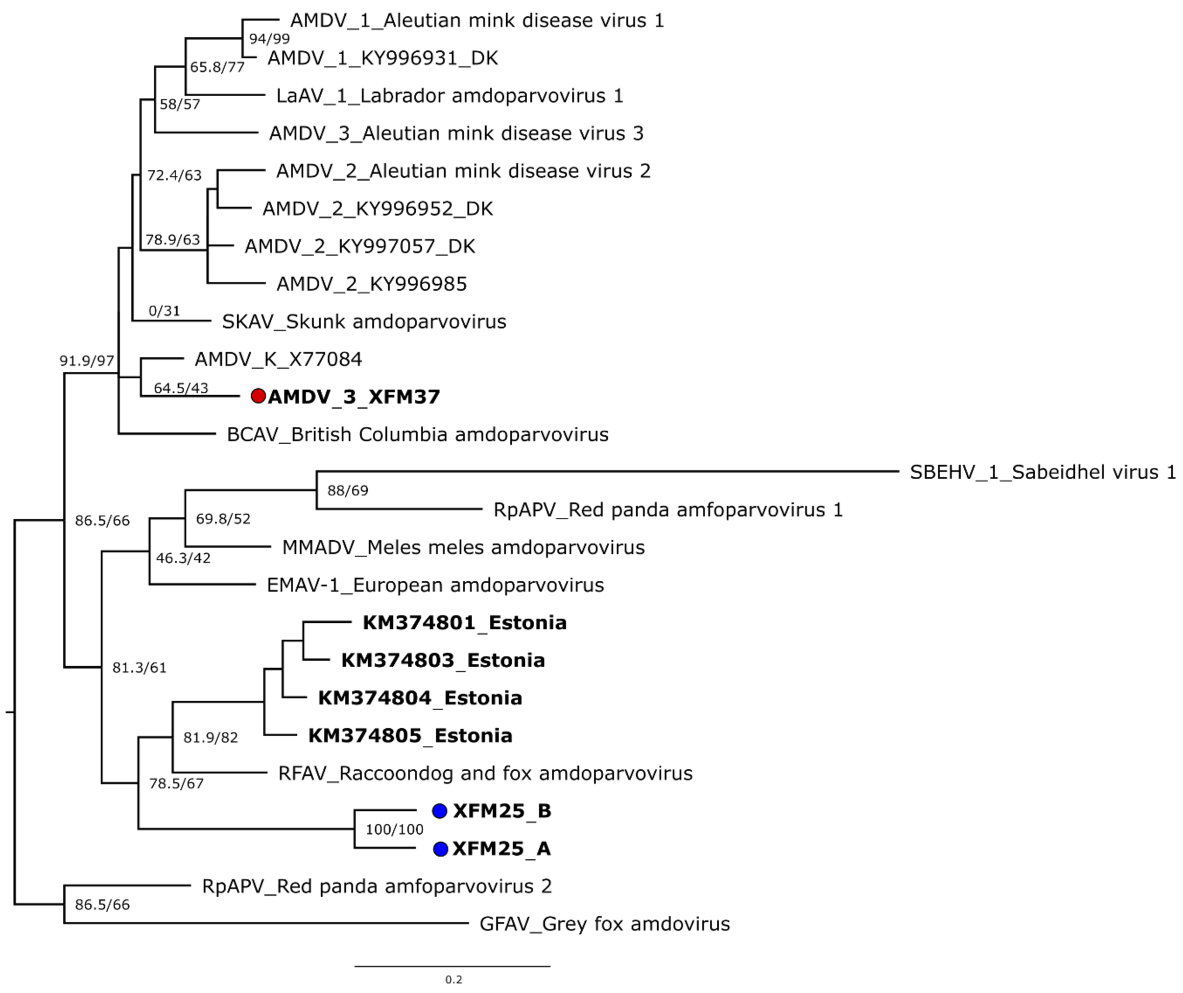

Supplementary Figure S2. Phylogenetic analysis comparing study viruses to those found in ferrets.

The maximum likelihood tree was constructed using partial VP nucleotide sequences (~370 nt) from representative amdoparvoviruses, including known AMDV-1, AMDV-2, and AMDV-3 variants, as well as other carnivoran-associated amdoparvoviruses. The tree was inferred using the TVM+F+I+G4 substitution model in IQ-TREE2, with branch support assessed by 1,000 ultrafast bootstrap replicates and SH-aLRT tests. The newly identified viruses from Denmark (XFM and FFM strains) are shown in bold and labelled with a colored circle. Viruses identified in ferrets are highlighted in red. The tree was rooted using the sequences within the non-Musteloidea clade.

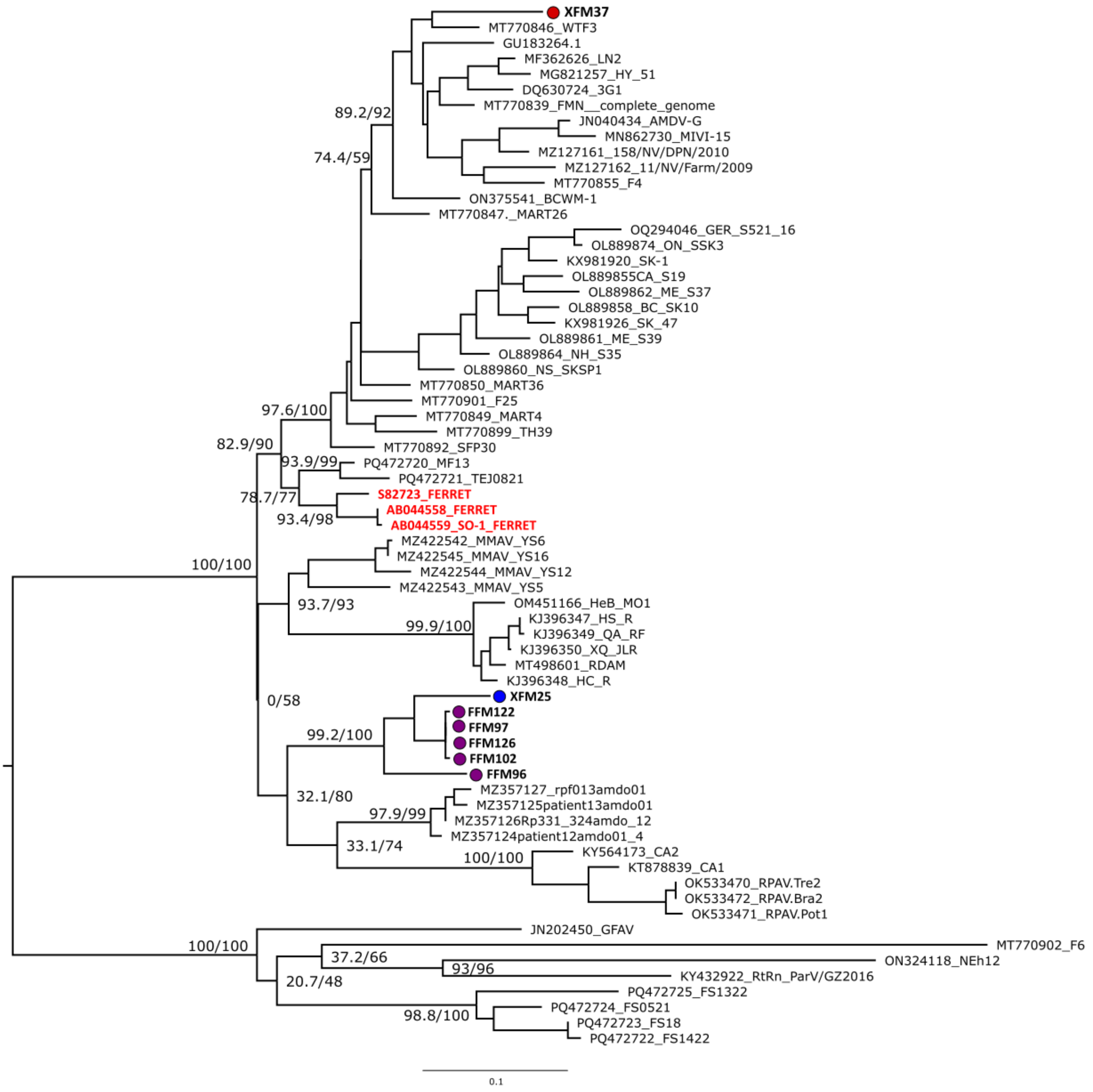

References:

Alex CE, Kubiski SV, Li L, Sadeghi M, Wack RF, McCarthy MA, et al. Amdoparvovirus infection in red pandas (*Ailurus fulgens*). Vet Pathol. 2018;55(4):552–61.

Canuti M, Doyle HE, P Britton A, Lang AS. Full genetic characterization and epidemiology of a novel amdoparvovirus in striped skunk (*Mephitis mephitis*). Emerg Microbes Infect. 2017;6(5):e30.

Canuti M, McDonald E, Graham SM, Rodrigues B, Bouchard É, Neville R, et al. Multi-host dispersal of known and novel carnivore amdoparvoviruses. Virus Evol. 2020a;6(2):veaa072.

Canuti M, Todd M, Monteiro P, Van Osch K, Weir R, Schwantje H, et al. Ecology and infection dynamics of multi-host amdoparvoviral and protoparvoviral carnivore pathogens. Pathogens. 2020b;9(2):124.

Canuti M, Pénzes JJ, Lang AS. A new perspective on the evolution and diversity of the genus *Amdoparvovirus* (family *Parvoviridae*) through genetic characterization, structural homology modeling, and phylogenetics. Virus Evol. 2022;8(1):veac056.

Canuti M, Mira F, Villanúa D, Rodríguez-Pastor R, Guercio A, Urra F, et al. Molecular ecology of novel amdoparvoviruses and old protoparvoviruses in Spanish wild carnivorans. Infect Genet Evol. 2025;128:105714.

Gottschalck E, Alexandersen S, Storgaard T, Bloom ME, Aasted B. Sequence comparison of the non-structural genes of four different types of Aleutian mink disease parvovirus indicates an unusual degree of variability. Arch Virol. 1994;138(3-4):213-31.

Hagberg EE, Pedersen AG, Larsen LE, Krarup A. Evolutionary analysis of whole-genome sequences confirms inter-farm transmission of Aleutian mink disease virus. J Gen Virol. 2017;98(6):1360–71.

Kamani J, González-Miguel J, Msheliza EG, Goldberg TL. Straw-Colored fruit bats (*Eidolon helvum*) and their bat flies (*Cyclopodia greefi*) in Nigeria host viruses with multifarious modes of transmission. Vector Borne Zoonotic Diseases. 2022;22(11):545-52.

Li L, Pesavento PA, Woods L, Clifford DL, Luff J, Wang C, et al. Novel amdovirus in gray foxes. Emerg Infect Dis. 2011;17(10):1876–8.

Shao XQ, Wen YJ, Ba HX, Zhang XT, Yue ZG, Wang KJ, et al. Novel amdoparvovirus infecting farmed raccoon dogs and arctic foxes. Emerg Infect Dis. 2014;20(12):2085–8.

Wu Y, Zhao Y, Zhang X, Wei T, Peng Q, Wang J, et al. Diverse amdoparvoviruses infection of farmed Asian badgers (*Meles meles*). Arch Virol. 2024;169(7):139.

Zhao M, Yue C, Yang Z, Li Y, Zhang D, Zhang J, et al. Viral metagenomics unveiled extensive communications of viruses within giant pandas and their associated organisms in the same ecosystem. Sci Total Environ. 2022 May 10;820:153317.
